# The Landscape of Primary Gastric Leiomyosarcoma in Texas Population: Analysis of Texas Cancer Registry Data

**DOI:** 10.7759/cureus.49403

**Published:** 2023-11-25

**Authors:** Tengfei Wang, Riyam Zreik, Bing Leng

**Affiliations:** 1 Pathology, Baylor Scott & White Health, Temple, USA

**Keywords:** bmi, seer summary stage, tcr, leiomyosarcoma, stomach

## Abstract

Introduction

Primary gastric leiomyosarcoma is an extremely rare disease. There have been no previous studies regarding gastric leiomyosarcoma in the Texas population.

Methods

Anonymous data of gastric leiomyosarcoma from the Texas Cancer Registry (TCR) was used. Information collected included the primary tumor site, age at diagnosis, gender, race/ethnicity, diagnosis and treatments, tumor size, lymph node and metastasis status, grade and stage, body weight and height, public health regions and payer, metropolitan status, neighborhood poverty level, smoking status, survival interval, and cause of death for statistical analysis.

Result

Thirty-three cases from 2003-2019 were selected. Primary gastric leiomyosarcoma was more commonly diagnosed in patients over 50 years of age, females, and individuals of white race. The diagnosis was primarily based on histology, and the disease was typically treated with surgery and chemotherapy. At the time of diagnosis, 45.5% of patients were in the late stage, and 48.5% of patients died from gastric leiomyosarcoma with a mean survival interval of 15.3 months. BMI scores showed a positive correlation with survival intervals. Surveillance, Epidemiology and End Results (SEER) tumor staging was associated with the prognosis of gastric leiomyosarcoma.

Conclusion

There were multiple disparities among patients with primary gastric leiomyosarcoma in the Texas population. The SEER summary stage was associated with the prognosis of gastric leiomyosarcoma.

## Introduction

According to the World Health Organization classification, the diagnosis of primary gastric leiomyosarcomas is so rare in the post-gastrointestinal stromal tumors (GISTs) era that there is a lack of data on its demographic and clinicopathological features [1]. Specific immunomarkers for GIST, such as CD117(c-kit), CD34, and DOG1, were introduced in the late 1990s. Currently, the diagnosis of primary leiomyosarcoma integrates histologic features, the presence of smooth muscle antigens, and the absence of GIST immunomarkers [2].

Most publications related to gastric leiomyosarcoma are based on limited case numbers. A recent comprehensive literature search [3] identified only 19 cases of primary gastric leiomyosarcomas with a convincing diagnosis. Nevertheless, gastric leiomyosarcoma usually originates from the muscularis propria and muscularis mucosa layers [4]. Tumors are located in the gastric body, fundus, antrum, cardia, and pylorus [3]. The documented tumor size varies from 1 to 18 cm [5], and these tumors have been observed in patients aged between 16 and 74 years, with roughly similar risk for men and women (11 males vs. 8 females) [3].

Possible prognostic factors for gastric leiomyosarcomas include histopathological grade and type, tumor size, synchronous metastasis, and parietal gastric infiltration [6,7]. Mitosis was initially considered not to be a prognostic factor [1]. Still, a recent large cohort study revealed that extraesophageal intramural smooth muscle tumors, such as those in the stomach, small intestine, and colorectum, measuring larger than 10 cm and with ≥3 mitoses per 5 mm^2^, had a more aggressive behavior and a poor prognosis. As a result, the authors proposed defining leiomyosarcoma vs. leiomyoma by using tumor size and mitotic activity. This study included 180 gastric smooth muscle tumors, although no detailed clinicopathological data were available [8].

Here, we utilized anonymous data provided by the TCR to study primary gastric leiomyosarcoma in Texas. We analyzed primary sites of tumor, age of diagnosis, sex and race/ethnicity, diagnosis and treatments, tumor size, lymph node and metastasis, grade and stage, body weight and heights, public health regions and payer, metropolitan status and poverty level, smoking status, survival interval, and death cause. Our analysis revealed multiple disparities in gastric leiomyosarcomas among the local population in Texas.

## Materials and methods

This study was approved by the Baylor Scott and White Health Ethics Committee. Anonymous cancer incidence data in the period of 1995-2019 were obtained from the TCR, which is funded by the National Cancer Institute’s SEER and certified by the North American Association of Central Cancer Registries (NAACCR).

The primary site of the cancer and its histology in the TCR were coded using the International Classification of Diseases for Oncology Third Edition (ICDO-3) [9]. We collected data for gastric leiomyosarcoma with a primary site coded as C16.0-C16.6, C16.8-16.9, and histology code 8890. Texas population data were collected from the U.S. Census Bureau [10].

We retrieved the following data: primary sites of tumor, age of diagnosis, sex and race, diagnosis and treatments, tumor size, lymph node and metastasis, grade and stage, body weight and height, public health regions and payer, metropolitan status and neighborhood poverty level, smoking status, survival interval, and death cause. The data interpretation was based on the TCR data dictionary 1995-2019 version.

Raw data was further analyzed using SAS version 9.4 (SAS Institute Inc., Cary, NC) and GraphPad Prism 6 (GraphPad, Boston, MA). We conducted unpaired t-test, Pearson’s correlation coefficient, one-way ANOVA test, and Tukey's HSD test for post-hoc analysis [11]. BMI was calculated using the formula: BMI = [Weight (lbs) / Height (inches)^2^] x 703. The Log-rank (Mantel-Cox) test and plot Kaplan-Meier survival curve were used to estimate the survival rate [12]. We considered death from gastric leiomyosarcoma as the events, alive or death from other diseases as censors.

## Results

The unadjusted incidence rate of primary gastric leiomyosarcoma (1995-2019) in Texas

**Figure 1:**
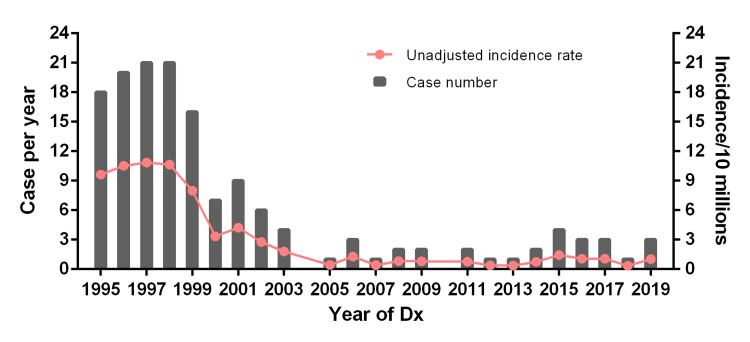
Crude incidence rate and case numbers from 1995-2019 in Texas population.

The crude incidence rate of primary gastric leiomyosarcoma in Texas ranged from 2.8 to 10.8 cases per 10 million population during the period of 1995-2002. However, the crude incidence rate decreased to 0.35-1.82 per 10 million population from 2003 onwards, as shown in Figure 1.

The yearly case numbers decreased from 6~21 cases (1995-2002) to 0~4 cases (2003-2019).

Thirty-three cases (2003-2019) were collected in this study

Given that the definitive diagnosis of gastric GISTs vs. leiomyosarcomas was established in the late 1990s, and gastric leiomyosarcoma became rare thereafter, we hypothesized that the incidence rate of gastric leiomyosarcoma would remain consistently low each year in the 2000s. Therefore, we chose the year 2003 as a cut-off to collect cases for further analysis in this study. There have been a total of 33 cases of primary gastric leiomyosarcoma documented in the TCR dataset from 2003 to 2019.

Patients’ age at diagnosis, sex, and race/ethnicity

The patients were all adults, ranging from 28 to 89 years old at the time of diagnosis, with a mean age of 56.6 and a median age of 66 years. Most patients were over 50 years old, including 16 (48.5%) patients between the ages of 51 and 75, and 11 (33.3%) cases over 75 years old. Notably, a subgroup of 6 (18.2%) patients were 50 years old or younger, with a mean age of 38.5 and a median age of 35.5 years.

**Figure 2:**
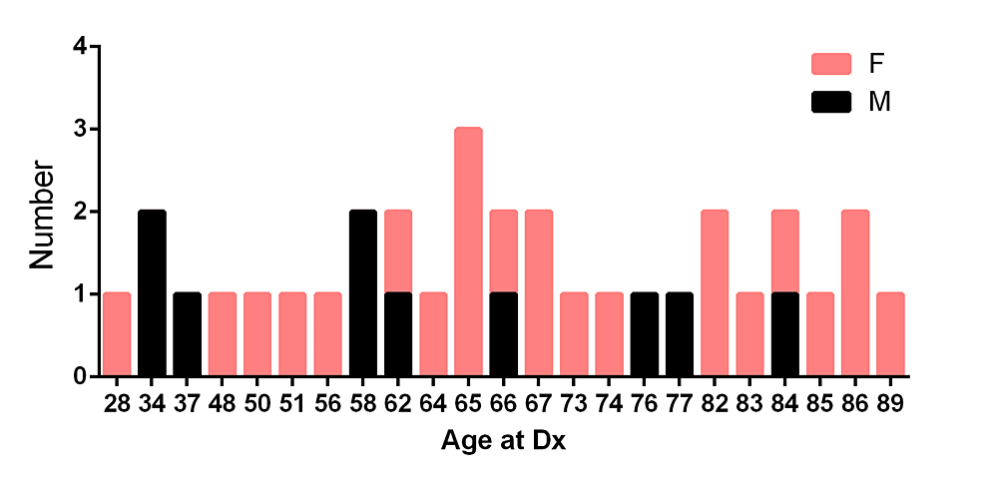
Sex difference of gastric leiomyosarcoma cases at age of diagnosis in Texas.

The overall female-to-male ratio was 2.3, with 23 females and 10 males. The distribution pattern (Figure 2) indicates that females were more commonly affected by this tumor. Particularly, there were 51.5% (17/33) white females, 24.2% (8/33) white males, 9.1% (3/33) African American females, 3.0% (1/33) African American males, 6.1% (2/33) Asian American (Chinese and Thai) females, 3.0% (1/33) Asian American (Japanese) males and 3.0% (1/33) other race females.

Among these 33 cases, 75.8% were White (25/33), followed by African American at 12.1% (4/33), and Asian American at 9.1% (3/33), including one patient each of Chinese, Japanese, and Thai descent.

The TCR also collected data regarding Hispanic/Latino ethnicity, identifying five (15.2%) Hispanic/Latino patients, including three Hispanic/Latino females and two males. The majority (4/5) were of Mexican origin.

Tumor site, tumor size, positive lymph nodes, distant metastasis, AJCC TNM stage, NAACCR grade and SEER summary stage, diagnosis and treatment methods

The tumor sites include the gastric fundus (7/33, 21.2%), greater curvature (6/33, 18.2%), gastric corpus (4/33, 12.1%), pyloric antrum (3/33, 9.1%), and lesser curvature (3/33, 9.1%). One case (1/33, 3.0%) was located in the gastric cardia, and another (1/33, 3.0%) was an overlapping lesion of the stomach. The remaining cases (8/33, 24.2%) were at not otherwise specified (NOS) sites. Tumors in the three most common sites were more frequent in the white population, among females, and had high mortality (see Table 1).

**Table 1:** Primary gastric leiomyosarcoma cases of different sites, races, sex, age of diagnosis, and prognoses in Texas. n: case number; NOS: Not otherwise specified; AA: African American

Tumor Location	n	Alive (n)	Age (mean, year)	White (n)	AA (n)	Female
Cardia	1	0	58	1	0	0.0%
Gastric fundus	7	2	63	4	2	71.4%
Gastric corpus	4	1	77	3	0	100.0%
Pyloric antrum	3	0	70	3	0	33.3%
Lesser curvature	3	0	61	2	1	100.0%
Greater curvature	6	2	56	6	0	50.0%
Overlapping lesion	1	1	67	1	0	100.0%
Gastric, NOS	8	2	71	5	1	75.0%

Twenty-three cases reported varying tumor sizes, ranging from 1.0-2.0 cm to 19.0 cm. The mean size of tumors (22 cases after excluding a case of 1.0-2.0 cm) is 7.8 cm, with a median of 7.3 cm.

The regional lymph node status was mostly unknown (24/33), with 17 cases not examined and seven cases not recorded. In addition, there were seven cases with negative lymph nodes and two with positive lymph nodes.

Five patients were reported with unknown distant metastasis records (at least M0). Distant metastatic status was reported in 14 patients including eight subjects with no distant metastasis (M0) and six cases with distant metastasis (M1). These six cases included four cases categorized under "Distant metastasis except for distant lymph node(s), Carcinomatosis, metastasis to ovary(ies), positive peritoneal cytology". Additionally, one case had distant metastasis along with distant lymph node(s), and another case had distant metastasis, but no further information was available.

Only three cases had American Joint Committee on Cancer (AJCC) cTNM staging (one case had both cTNM and pTNM). Regarding the tumor grade, 18 cases had unknown grades. There were six cases classified as NAACCR grade IV, followed by grade III in six cases, and grade I in three cases.

SEER summary stage of the tumor was reported in 27 patients. About 21.2% (7/33) of cases were localized tumors (stage 1), four cases were regional due to direct extension only, one case was regional due to regional lymph node metastasis only, and 45.5% (15/33) of cases had distant metastasis or systemic disease (stage 7). Six cases did not report their staging.

Patients were diagnosed mainly by histology (93.9%, 31/33). Other diagnostic methods include cytology (1/33) and radiography/imaging (1/33).

The treatment plan was summarized from each treatment and the treatment sequence. Treatment included surgery, radiation therapy, and chemotherapy with single or multiple agents. About 39.4% of patients (13/33) were treated with surgery only, 24.2% of patients (8/33) were treated with chemotherapy alone, and 9.1% of patients (3/33) used surgery followed by chemotherapy strategy. One patient had surgery before radiation, one patient had chemotherapy before surgery, one patient was treated with both chemotherapy and radiation, and one patient had chemotherapy before and after surgery. Additionally, two patients did not receive any treatment, and three patients did not report their treatment plans.

Public health regions, metropolitan areas, neighborhood poverty, payer at diagnosis, smoking status, and BMI score

**Figure 3:**
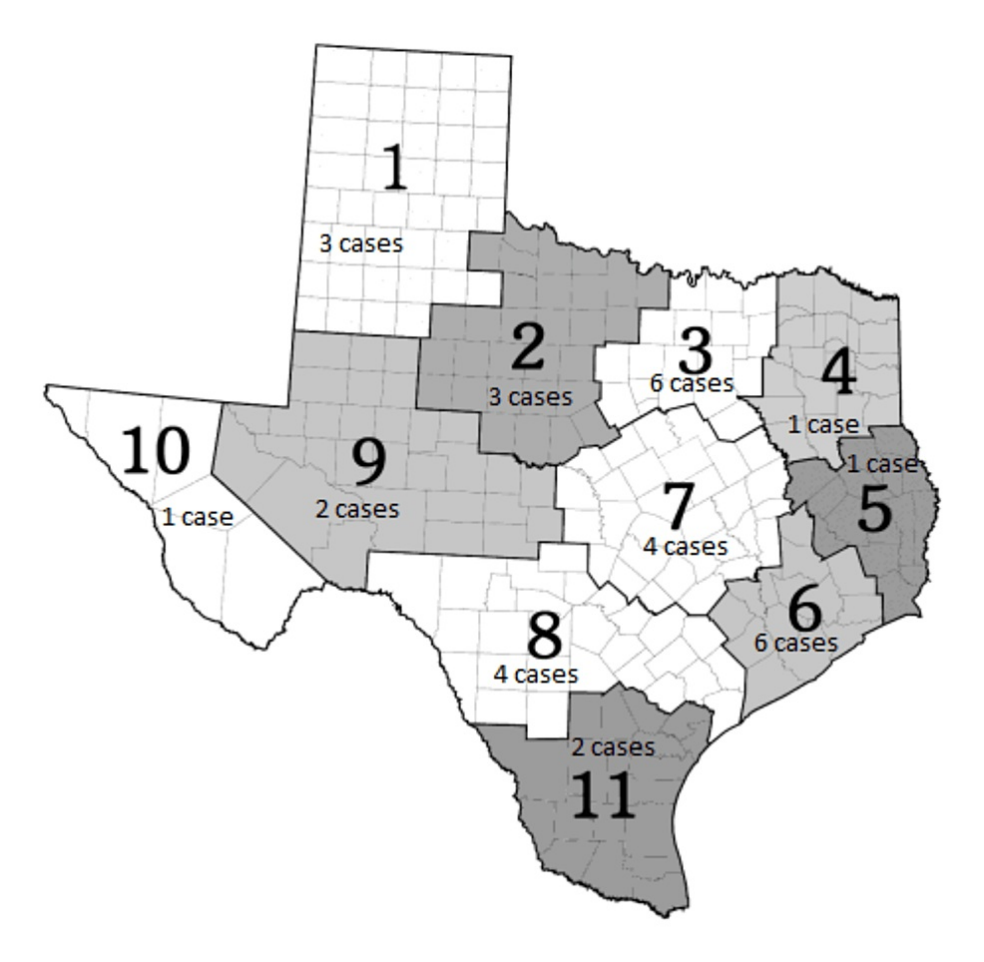
Primary gastric leiomyosarcoma case distributions in TX public health regions. Bold digits are Public Health Regions (PHR) numbers.

These 33 patients lived in 28 (out of 254) counties in Texas, distributed across 11 Public Health Regions (PHR) (Figure 3) [13].

These 33 patients came from different rural/urban areas. Fourteen patients (42.4%) were from counties in metropolitan areas (metro areas) with a population of 1 million or more. Six cases (18.2%) were from counties in metro areas with a population of 250,000 to 1 million, followed by five cases (15.2%) each from counties in metro areas with fewer than 250,000 in population and urban areas with a population of 2,500 to 19,999, which are adjacent to a metro area. To simplify, eight patients lived in non-metro areas, while 25 patients lived in metro areas.

Neighborhood poverty levels of the patients were based on the U.S. Census and American Community Survey. Seven patients lived in areas with 20-100% poverty (21.2%, 7/33, high-poverty neighborhoods [14]), and 14 patients lived in areas with 10-19.9% poverty. Six patients were in areas with 5-9.9% poverty and six in 0-5% poverty.

Regarding primary payer at diagnosis, 15 patients (45.5%, 15/33) used Medicare, four cases used private insurance, two patients each used Medicaid and Tricare, one case was not insured, and one used military insurance. Eight cases had unknown insurance status.

Sixteen patients reported their consumption of tobacco products, including cigarettes, pipes, cigars, chewing tobacco, snuff, and others. Sixty-nine percent of patients (11/16) never used tobacco products, and 19% of patients (3/16) were current users. Twelve percent of patients (2/16) were former users but quit more than one year.

BMI scores were calculated for 17 patients who reported weights and heights. BMI scores under 20 are considered underweight, 20 to 24.9 are considered normal, 25 to 29.9 are overweight, 30 to 34.9 are obese, and scores above 35 are extremely obese. Among these 17 patients, three (17.6%, 3/17) were underweight, five (29.4%) had a normal BMI, three (17.6%) were overweight, five (29.4%) were obese, and one (5.8%) was extremely obese. In short, 11 patients were non-obese, while six patients were obese.

Survival interval analysis and estimation of survival rate

Among those 33 patients, eight (24.2%) were alive at the last contact. Sixteen (48.5%) patients died from gastric leiomyosarcoma, i.e., 11 (33.3%) stomach malignancies and five (15.2%) connective and soft tissue malignancies. In contrast, other causes of death included three (9.1%) cases of duodenum or ill-defined sites malignancies; three (9.1%) cases of melanoma, breast cancer, or lung cancer; three (9.1%) cases of acute myocardial infarction, pneumonia, or chronic obstructive pulmonary disease.

For those 16 patients who died from gastric leiomyosarcoma, their survival intervals ranged from 1 to 48 months. For the eight living patients, their survival time ranged from 17 months to 203 months. In addition, for those who died of other causes, their survival intervals ranged from 5 months to 117 months. The average survival months are significantly different between the alive group, the group dead from leiomyosarcoma, and the group dead from other diseases (one-way ANOVA, F(2,30)=11.09, p = 0.0002). Tukey’s test for multiple comparisons found that significant differences were present in the comparison of the alive group vs. the group dead from leiomyosarcoma (p=0.0002), and the alive group vs. the group dead from other diseases (p=0.0063). These findings, together with SEER stage findings, indicated that primary gastric leiomyosarcoma can be aggressive.

We also analyzed the correlation of BMI score, tumor size, and age at diagnosis with survival intervals. Among 16 patients who died from gastric leiomyosarcoma, there were 11 cases (survival time ranges 2-29 months) having BMI scores. Surprisingly, our analysis showed that BMI score was positively correlated with survival intervals of gastric leiomyosarcoma (Pearson correlation coefficient of r=0.71, p=0.015). In contrast, tumor size (r=-0.042, p=0.85) and age (r=-0.056, p=0.87) were not correlated with survival intervals.

We then stratified patients by using tumor site, age (≤50 years vs. 51-75 years vs. >75 years), sex, race (White vs. African American vs. Asian), grade, distant metastasis, positive regional node numbers, SEER summary stage, treatment plan, smoking status, metro area, primary payer, neighborhood poverty levels and PHR. There was no difference in average survival time between subgroups based on variables (p values > 0.05). Due to the very limited data in each subgroup, a larger sample size is warranted to confirm these findings.

**Figure 4:**
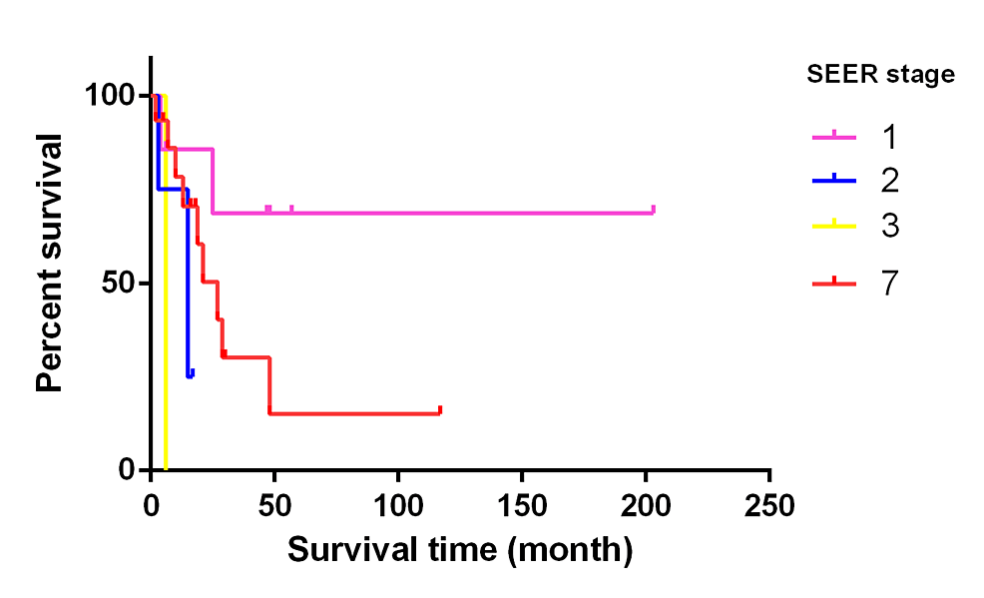
Kaplan-Meier survival curve in gastric leiomyosarcoma patients with different SEER summary stages. Log-rank p = 0.024

**Figure 5:**
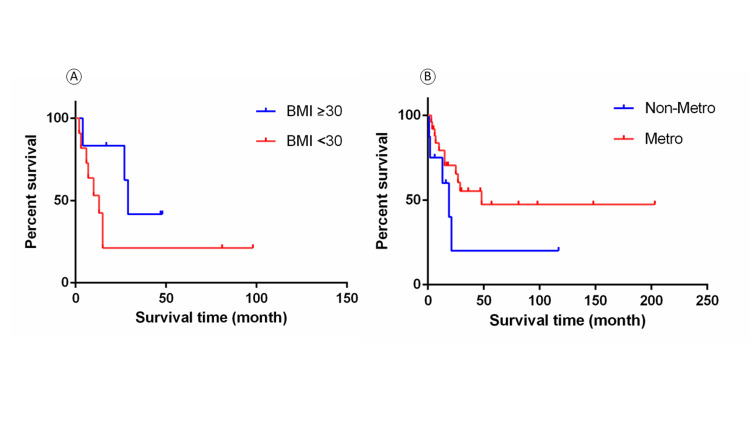
Kaplan-Meier survival curves in different BMI score (A) and Metro area (B) groups with gastric leiomyosarcoma. Log-rank p-values >0.05

Furthermore, we estimated the survival rate by using the Log-rank (Mantel-Cox) test and plotted Kaplan-Meier survival curve. We considered death from gastric leiomyosarcoma as the events, otherwise as censors. The same factors listed above were evaluated. Our results showed that SEER summary stage of gastric leiomyosarcoma was associated with prognosis (Log-rank p = 0.024), with patients in the late stage trending toward worse outcomes (Figure 4). BMI score <30 vs. ≥30 (or non-obese vs. obese, Log-rank p = 0.17) and metro area (metro vs. non-metro, Log-rank p = 0.12) were not considered prognostic, but a potential trend can be seen in the survival curve (Figure 5). Other factors were not related to the prognosis of gastric leiomyosarcoma. Additionally, there was no difference in the means of BMI score, tumor size, or age (p > 0.05) when stratified by prognosis (i.e., events vs. censors).

If only considering the 11 patients who died from stomach malignancies as gastric leiomyosarcoma mortality, the sample size for subgroups became extremely small. However, the above conclusions remain unchanged.

## Discussion

In this study, we found multiple disparities among gastric leiomyosarcoma patients in Texas. Primary gastric leiomyosarcoma was more common in patients older than 50 years. The ratio of female to male patients was 2.3. The most affected race was white. The tumor was mainly diagnosed by histology and treated with surgery and chemotherapy. About 45.5% of diagnosed patients were in the late stage, and 48.5% of patients died from gastric leiomyosarcoma with a mean survival interval of 15.3 months. Tumor SEER staging was associated with prognosis.

Primary gastric leiomyosarcoma is rare, and its etiology remains unclear. *Helicobacter pylori* bacterial infection could be a potential risk factor since it causes other gastric malignancies, such as adenocarcinoma and mucosa-associated lymphoid tissue (MALT) lymphoma [15]. Neighborhood poverty, defined as the percentage of families whose income in the past 12 months was below the poverty level [16], has a significant causal link to health outcomes [14], and is associated with cancer risk burden [17], or colon cancer survival [18]. The potential causes underlying neighborhood poverty include food deserts [19] and air toxics [17, 20]. Further investigation is required to study the relationship between food deserts or air toxics and gastric leiomyosarcoma since approximately 1/5 of our patients lived in high-poverty neighborhoods.

We found some unique features among primary gastric leiomyosarcoma patients in Texas. For example, 18.2% of patients were 50 years old or younger, with a mean age of 38.5 and a median age of 35.5 years. Men and women did not have an equal risk for this disease; instead, more female patients were identified. Most tumors were located in the gastric fundus (21.2%) followed by greater curvature (18.2%) and gastric corpus (12.1%). Most patients were from the white population. Limited Hispanic/Latino cases (n=5) were identified in the records compared with the fact that the Hispanic population was the second-largest population in TX per U.S. Census Bureau.

Among the literature, gastric leiomyosarcomas can be identified by contrast-enhanced CT scan [6], MRI [21], endoscopy, and endoscopic ultrasound [22]. However, pathology is the definitive diagnosis method. In our study, most cases were diagnosed by histology.

Surgical resection is the current treatment of choice for gastric leiomyosarcoma [5], and if metastasized to other organs, chemotherapy, chemoembolization, and high-intensity focused ultrasound treatment can be a management option [5,23] per the published literature. Here, we find that surgery or chemotherapy alone was commonly used in Texas patients. Also, some patients were treated with combined chemo/radiation/surgery or palliative management.

Our results showed that SEER summary stage was related to prognosis, i.e., different prognosis with SEER stage 1 vs. 7. More data are warranted to confirm stage 2 and 3 survival curves. However, our results did not support a prognostic effect of other factors, i.e., age, sex, race, grade, distant metastasis, positive regional node numbers, tumor size, treatment plan, smoking status, poverty levels, and PHR. Although BMI score and metro area were not considered as prognostic factors, a potential trend can be seen in the survival curve. Interestingly, a positive correlation was revealed between BMI scores and survival times. Moreover, the finding that obesity is related to survival has also been reported in lung cancer, renal cell carcinoma, and melanoma [24].

This study has several limitations. Firstly, the dataset consists of a small number of cases, and to enhance the robustness of our conclusions, more data is needed. However, due to the rarity of this disease, obtaining a larger dataset about the Texan population is not feasible at this time. Secondly, the dataset lacks information on the Fédération Nationale des Centres de Lutte Contre le Cancer (FNCLCC) system for grading gastric leiomyosarcoma and provides limited data on AJCC stages [25]. Additionally, the dataset has no data on *Helicobacter pylori* infection. Those are areas of interest in this rare disease.

## Conclusions

Our findings revealed multiple disparities among gastric leiomyosarcoma patients in Texas. Most cases involved individuals aged older than 50, with a higher proportion of female patients. The majority of tumors were located in the gastric fundus, and a significant portion of patients were from the white population. Treatment options included monotherapy with surgery or chemotherapy. Additionally, we found that the SEER summary stage was associated with the prognosis of gastric leiomyosarcoma.
